# The role of carcinoembryonic antigen-related cell adhesion molecule 1 in cancer

**DOI:** 10.3389/fimmu.2023.1295232

**Published:** 2023-11-24

**Authors:** Lisa Götz, Uwe Rueckschloss, Gözde Balk, Verena Pfeiffer, Süleyman Ergün, Florian Kleefeldt

**Affiliations:** ^1^ Institute of Anatomy and Cell Biology, Julius‐Maximilians‐University Würzburg, Würzburg, Germany; ^2^ Harvard Stem Cell Institute, Department of Stem Cell and Regenerative Biology, Harvard University, Cambridge, MA, United States

**Keywords:** CEACAM1, CEA, cancer, tumor, malignancy, metastasis, signaling

## Abstract

The Carcinoembryonic antigen-related cell adhesion molecule 1 (CEACAM1), also known as CD66a, is a member of the immunoglobulin superfamily. CEACAM1 was shown to be a prognostic marker in patients suffering from cancer. In this review, we summarize pre-clinical and clinical evidence linking CEACAM1 to tumorigenicity and cancer progression. Furthermore, we discuss potential CEACAM1-based mechanisms that may affect cancer biology.

## 1 Introduction

The glycoprotein Carcinoembryonic antigen (CEA)-related cell adhesion molecule 1 (CEACAM1, also CD66a) is a member of the immunoglobulin superfamily. CEACAM1 is expressed in a broad range of different tissues and cell types like epithelial cells, vascular cells, immune cells as well as cancer cells (1–3). Based on its downregulation in early stages of colorectal cancer (CRC) detected in some of the earlier studies CEACAM1 was initially regarded as a potential tumor suppressor (4). However, cumulative evidence from pre-clinical as well as clinical data suggests a more complex influence of CEACAM1 on cancer biology. For instance CEACAM1 affects anti-cancer immune reactions by modulating the function of natural killer cells and T-cells (3). Furthermore, later clinical association studies indicate that CEACAM1 expression seems more likely to be associated with cancer progression and poor prognosis in most cancer entities. Therefore, the concept of CEACAM1 as a tumor suppressor needs to be revised. For that purpose, in this review we will I) summarize the clinical data on CEACAM1 and cancer progression and survival, II) give an overview of potential underlying mechanisms and III) highlight current therapeutic approaches and bottlenecks.

## 2 CEACAM1 structure and signaling

CEACAM1 is a member of the Carcinoembryonic antigen (CEA)-related cell adhesion molecule family. CEACAMs are either transmembrane proteins (CEACAM1, 3, and 4) or membrane-anchored G protein-coupled receptors (CEACAM 5, 6, 7, and 8). Their ectodomain consists of a variable number of IgGC2-related domains and a N-terminal IgV-related domain.

CEACAM1 comprises an extracellular, a transmembrane and a cytoplasmatic domain (5, 6). Due to alternative splicing 12 different variants exist in humans that differ in the composition of immunoglobulin (Ig)-like ectodomains as well as in the cytoplasmatic domain (7). The cytoplasmatic domain exists either in a long (CEACAM1-L, inclusion of exon 7) or a short (CEACAM1-S, exclusion of exon 7) version. Only CEACAM1-L possesses two immunoreceptor tyrosine-based inhibitory motifs (ITIMs) that when phosphorylated serve as a docking site for SRC homology 2 (SH2) domain-containing signaling proteins like SRC family kinases (SFKs) or cytoplasmic tyrosine phosphatases (SHPs). Thereby CEACAM1 can affect intracellular signaling in different ways (8–10). Besides formation of heterodimers with other CEACAM family members or serving as a pathogen receptor, CEACAM1 can either act as a monomer, a homodimer (i.e. two CEACAM1-L) or a heterodimer (CEACAM1-L and CEACAM1-S) (10–14). This is of particular interest because CEACAM1 monomers, homo- and heterodimers show different binding affinities for SFKs and SHPs (8). Thus, the expression ratio of CEACAM1-L to CEACAM1-S might also affect CEACAM1 signaling Unfortunately, so far only for breast cancer the L/S ratio of CEACAM1 was analyzed and found to be altered (15). Having such data for other cancer entities too might help to explain divergent results concerning cancer stage-related CEACAM1 expression and patient survival.

## 3 CEACAM1 in human cancer

Several studies investigated the potential impact of CEACAM1 on cancer in human specimen. Due to an observed downregulation of CEACAM1 in early CRC, CEACAM1 was originally suggested to be a tumor suppressor (16, 17). However, these studies have important limitations. First, they were based only on a small number of samples. Second, the control tissue was taken from the same specimen right beside the cancerous tissue. Since CEACAM1 is upregulated under inflammatory conditions the peri-tumor milieu might have already altered basal CEACAM1 expression in those controls (18). Finally, in both studies only mRNA expression was analyzed lacking information on the presence of the functional protein. In contrast, a later analysis by Kang et al. revealed upregulation of CEACAM1 protein expression in colon adenocarcinoma compared to adenoma in a larger cohort (19). Therefore, the initial hypothesis of CEACAM1 downregulation in CRC must be revised. This is in line with Ieda et al. who have shown that CEACAM1-L dominance associates with shorter survival time in patients (20). The same group reported that CEACAM1-S located at the invasion front of the primary lesion is associated with poor prognosis of patients with colorectal liver metastasis (21).

Besides CRC, several studies indicate a potential role of CEACAM1 in multiple cancer entities. The majority of these studies shows enhanced CEACAM1 expression with progressive tumor stage and/or metastasis. Moreover, in most studies enhanced CEACAM1 expression correlates with shorter overall patient survival. However, from those clinical studies at present it cannot be excluded that CEACAM1 upregulation might be a side effect rather than a driver of cancer progression and metastasis. As mentioned above, this could be due to the cancer-associated pro-inflammatory milieu that might promote CEACAM1 expression (18, 22). Therefore, to analyze the relation of cause and effect between CEACAM1 and cancer and the underlying signaling in pre-clinical cancer models is a prerequisite for a later transition of experimental findings into clinical applications.

To enable a better overview of different cancer entities clinical studies are summarized in **Table 1**. For proper interpretation one has to keep in mind that CEACAM1 expression was analyzed by different methods (e.g., RNA expression, histological staining) using specimen of different origin (e.g., serum concentration, staining of tissue slides).

**Table 1:** Correlation of CEACAM1 expression with cancer stage and patient survival.

Malignancy	Association of CEACAM1 expression with	
	cancer stage	patient survival
Breast cancer	- ^A^ (23) + ^A^ (24)	
Lung cancer	+ (25–28)	- (25, 28–30)
Colorectal cancer	-^B^ (16, 17) + (16, 17, 19)	-^C^ (20, 21)
Melanoma	+ (31–36)	- (37, 38)
Bladder cancer	-^D^ (39) + (40, 41)	
Pancreatic cancer	+ (42–46)	- (47)
Cervical cancer	+ (48)	
Prostate cancer	- (49–51)	
Esophageal/Larynx squamous cell carcinoma, Head and neck cancer, Oral cancer	+ (52, 53)	- (52, 54, 55)
Osteosarcoma	+ (56)	- (56)
Renal Cell carcinoma	+ (57, 58)	+ (58)
Thyroid cancer	+ (59, 60)	
Gastric cancer	- (61) +^E^ (62–64)	- (61)
Hepatocellular carcinoma	- (65) + (66)	-^F^ (66, 67) + (65)

This table summarizes clinical data regarding the correlation of CEACAM1 expression with cancer stage and patient survival. Cancer stage: decreased (-) vs. enhanced (+) CEACAM1 expression with progressive cancer stage. Patient survival: inverse (-) vs. positive (+) correlation of CEACAM1 expression and patient overall survival. Therefore - indicates that enhanced CEACAM1 expression goes along with shorter overall survival.

Please note that “CEACAM1 expression” was analyzed in different ways in the listed studies (e.g., RNA or protein in blood or tissue specimens). For reasons of clarity full information is not depicted in the table. However, some information are indicated for reasons of understanding. For further information please refer to the original data.

^A^ Data was reported by the same group within the same year with at least somehow divergent results: reduced CEACAM1 in breast cancer tissues compared with noncancerous breast tissues but enhanced CEACAM1 serum concentrations in the malignant group compared to healthy control group. It is not clear why these observations were not published together.

^B^ Nollau et al.: Data only for early stage adenoma (RNA).

^C^ Yamaguchi et al.: Recurrence of CRC liver metastasis after hepatectomy.

^D^ CEACAM1 is upregulated in endothelial cell adjacent to tumor tissue.

^E^ Shi et al.: Association with lymph nodes metastasis and TNM stage.

^F^ Zhu et al.: Relapse-free survival after curative resection of hepatocellular carcinoma.

Kiriyama et al.: Depending on CEACAM1 L-/S-Form ratio.

## 4 CEACAM1-dependent mechanisms in cancer

**Figure 1:**
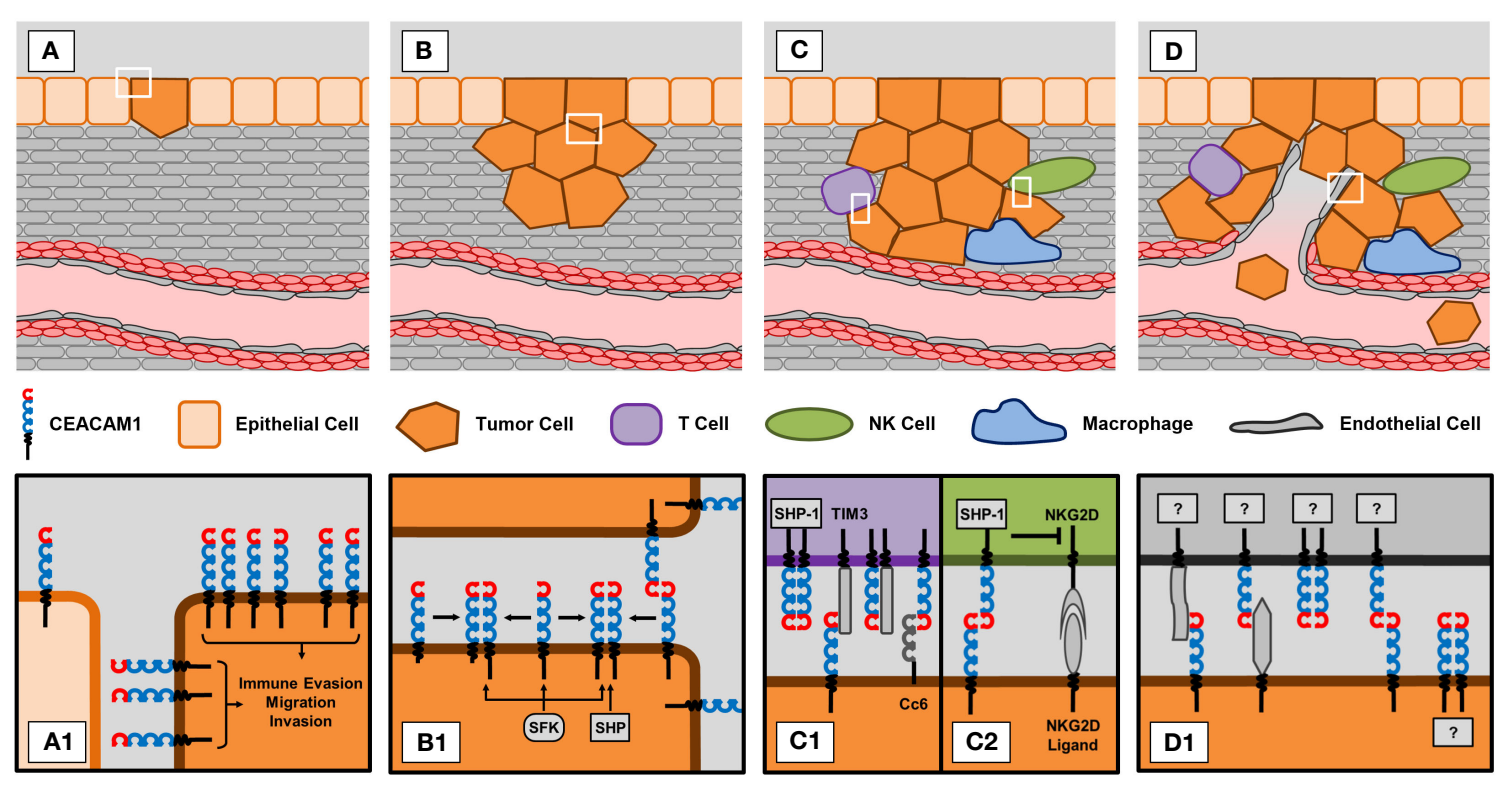
CEACAM1 in different stages of tumorigenesis and progression. **(A)** Epithelial-to-mesenchymal transition (EMT). **(A1)** Tumor cells show increased expression of CEACAM1 compared to normal epithelial cells. This upregulation of CEACAM1 is thought to promote processes that support tumor development, i.e. immune evasion, migration and invasion. **(B)** Proliferation of tumor cells. **(B1)** CEACAM1-dependent signaling greatly depends on the status of CEACAM1 molecules. Trans ligation of CEACAM1 molecules on neighboring cells also promotes cis ligation of CEACAM1 molecules expressed on the same cell. Depending on the expression ratio of long (L) and short (S) isoforms, the resulting dimers can consist of two CEACAM1-L molecules or of a combination of CEACAM1-L and CEACAM1-S. Whereas SHP phosphatases preferentially bind to CEACAM1-L homodimers, SFK kinases bind equally well to dimers and monomers. **(C)** Immune evasion. T cells and NK cells are capable of eliminating tumor cells. However, tumor cells have developed CEACAM1-dependent mechanisms to evade this immune response. **(C1)** Trans or cis ligation of CEACAM1 to TIM3 or binding of CEACAM6 (Cc6) to CEACAM1 on T cells abrogates anti-tumor activity of T cells. **(C2)** Similarly, trans ligation of CEACAM1 on tumor and NK cells blocks the cytotoxic effect of the activated NKG2D receptor expressed on NK cells. **(D)** Tumor vascularization. **(D1)** CEACAM1 is expressed on endothelial and tumor cells. Since CEACAM1 promotes angiogenesis, it is reasonable to speculate that interactions between endothelial and tumor cell CEACAM1 with or without involvement of other molecules might also affect tumor vascularization. However, this still needs to be investigated in more detail.

Despite its well-known association with cancer less is known about CEACAM1-dependent mechanisms that might affect cancer biology. To provide an overview we will summarize the current knowledge and discuss reasons that may account for the conflicting results reported in the literature regarding CEACAM1 and cancer cells. **Figure 1** summarizes the role of CEACAM1 in tumor biology.

### 4.1 Tumorigenesis and proliferation

Leung et al. showed that azoxymethane-mediated induction of colon cancer was aggravated in Ceacam1^-/-^ mice compared to WT mice suggesting an anti-tumorigenic effect of CEACAM1 expression (68). In line with that, CEACAM1 was also shown to repress epithelial to mesenchymal transition that is a critical step in oncogenesis (69).

Conflicting results have been reported regarding the effect of CEACAM1 on cancer cell proliferation that critically promotes tumor growth and size. Some studies reported inhibition of proliferation by CEACAM1. Overexpression of CEACAM1-L in MCF-7 breast cancer cells reduced EGF-stimulated cell growth (70). Similarly, CEACAM1 overexpression significantly suppressed proliferation of U266 and RPMI8266 multiple melanoma cells. Unfortunately, the underlying signaling mechanisms were not analyzed in this study (71). Singer et al. have also shown that overexpression of CEACAM1-L in A549 lung carcinoma cells decreased cell proliferation (72). Finally, Luebke et al. reported slightly increased proliferation rates due to the loss of CEACAM1 expression in human prostate cancer specimens again indicating an inhibitory effect of CEACAM1 on cancer cell proliferation (49).

In contrast, Han et al. found that siRNA-mediated CEACAM1 knockdown decreased proliferation in HT29 colon carcinoma cells. (73). Similarly, Ortenberg et al. have shown that overexpression of the L-form of CEACAM1 promotes proliferation of 526mel melanoma cells in a SOX2-dependent manner *in vitro*. Consequently, compared to normal 526mel cells, CEACAM1-overexpressing cells generated tumors with larger volumes when injected into mice (37).

### 4.2 Immune evasion

The immune system is capable to eliminate cells that are foreign to the body or show genetic aberrations as found in tumor cells. Thereby the immune system is critical to prevent cancer. However, cancer cells can evade recognition and subsequent elimination by the immune system (74). This also applies to immune cell-based therapies e.g. by chimeric antigen receptor T cells (CAR T cells) (75) and results in unsatisfactory efficiency of these immune therapies in solid tumors (76). Therefore, novel approaches circumventing cancer cell-mediated inhibition of immune cells are required to unleash the full potential of anti-cancer immunotherapies. Since CEACAM1 affects the activation of T cells, B cells and NK cells in the context of cancer, it might be a promising target (2).

T cells play a critical role in adaptive immune responses. Although programmed cell death protein 1 (PD1) and cytotoxic T-lymphocyte-associated protein 4 (CTLA4) are well known immune checkpoints that guard against autoimmunity under physiological conditions, these immune checkpoints can also prevent the anti-cancer efficiency of endogenous or therapeutically applied exogenous modified T cells. Therefore, immune checkpoint inhibition is used clinically in anti-cancer therapies (75). However, not all patients benefit from immune checkpoint inhibition equally. This indicates additional mechanisms that are not covered by these treatments. In that context activation-induced expression of CEACAM1 has been shown to inhibit T cell function, particularly the long isoform (77–80). Upon dimerization CEACAM1-L recruits SHPs to the TCR/CD3 complex that dephosphorylate adjacent kinases or adaptor proteins thereby decreasing proliferation and activation of T cells (80–82). Contrary to this, CEACAM1-L was shown to decrease Fas-mediated apoptosis in T cells independent of ITIM phosphorylation via β-catenin signaling modulation (83). In the context of a colitis model, CEACAM1 was shown to inhibit the differentiation of naive T cells into Th1 but not Th2 cells (84). In contrast CEACAM1-S is rather suggested to promote activation of T cells (85, 86). This may be related to the absence of an ITIM motif in the short isoform (87).

Furthermore, the heterophilic *cis* interaction of CEACAM1-L with T cell-immunoglobulin and mucin-domain containing 3 (TIM3) results in T cell exhaustion and immune tolerance (88). A similar inhibitory effect was observed in CRC and neck squamous cell carcinoma by *trans* interaction of CEACAM1 on tumor cells with TIM3 on T cells (54, 89). Inhibition of CEACAM1 and TIM3 in CRC models synergistically stimulated the anti-tumor immune response. This is in line with observations in hematopoietic malignancies showing that blocking the CEACAM1-TIM3 interaction results in an attenuated NF-κB signaling (90). In a clinical study of patients with glioma the expression of CEACAM1 on T cells was negatively correlated with the Karnofsky score (91). This was attributed to an inhibitory effect of CEACAM1 on T cells. Similarly a recent study using clinical data and an *in vivo* CRC mouse model indicates that CEACAM1 marks a suppressive subset of intra-tumoral regulatory T cells (92).

In addition, *trans* interaction of CEACAM1 with CEACAM6 was reported in several entities of solid cancers. Interestingly, CEACAM6 inhibition enhanced the tumor-suppressive T cell function indicating a crosstalk of CEACAM1 with other CEA family members (93).

B cells are involved in anti-cancer immune responses by secretion of antibodies targeting tumor cell-specific antigens as well as recruitment and activation of other immune cells. The impact of CEACAM1 on B cells in cancer is still contradictory discussed (94). Khairnar et al. stated that CEACAM1 positively regulates B cell proliferation and survival via Syk-mediated NF-κB signaling (95). In contrast, others found that CEACAM1 co-localizes with the B cell receptor and prohibits cytokine production in a PI3K-dependent manner (96, 97). Further Greicius et al. showed that a CEACAM1-specific antibody induced the proliferation of mouse B cells in combination with IgM cross-linking (98). The underling signaling was not investigated. Interestingly, binding of Opa proteins of Neisseria gonorrhoeae to CEACAM1 induces apoptosis in B cells (99). Thereby CEACAM1 could lead to a decreased immune system activation. This was also shown for T cells in the context of Fusobacterium nucleatum. This is an anaerobic bacterium that is associated with several tumor entities and promotes tumorigenesis (100).

NK cells are cytotoxic lymphocytes of the innate immune system that contribute to immune surveillance in cancer. Cells express ligands of activating receptors located on NK cells (e.g. NKG2D). However, NK cell activation is prevented by ligation of major histocompatibility complex (MHC) class I molecules normally expressed on healthy cells to killer immunoglobulin-like receptors (KIRs) on NK cells. Since cancer cells often downregulate MHC I expression to evade recognition by T cells, this inhibitory signal is lost and the NK cell is activated (100). This results in the cytolysis of the malignant cells (101).

CEACAM1 is abundantly expressed on activated NK cells and functions as inhibitory co-receptor (101–104). Homophilic CEACAM1 interaction between NK cells and tumor cells blocks the initiation of cytolysis by NK cells via SHP1-dependent dephosphorylation of guanosine nucleotide exchange factor Vav1 (105). Furthermore, CEACAM1 expression on CRC cells decreases NK cell-mediated cytolysis by diminishing surface presentation of NKG2D ligands (106). This effect may be isoform-dependent. While CEACAM1-L decreases NK cell activity via downregulation of the NKG2D ligands MICA and ULBP2 by enhanced shedding, CEACAM1-3S increases the expression of NKG2D ligands (107). Clinical data show an upregulation of CEACAM1 in NK cells by hepatitis C virus (HCV) infection that was accompanied by reduced NK cell activity *in vitro* and in patients. Thereby CEACAM1 might facilitate chronification of HCV infection and transition to hepatocellular carcinoma (108).

Cancer stem cells (CSCs) are a special subtype of cancer cells, that are thought to multiply indefinitely and are resistant to chemotherapy. Epithelial cell adhesion molecule (EpCAM) is a well-established marker of CSCs (109). EpCAM expression in liver CSCs promotes resistance against NK cell-mediated cytotoxicity via upregulation of CEACAM1 (110).

Based on these findings, the impact of CEACAM1 inhibition on NK cell activity was investigated in pre-clinical studies. Antibody-mediated inhibition of CEACAM1 in head and neck squamous cell carcinoma cells enhances NK cell anti-cancer activity *in vitro* (111). Similar results were obtained in a non-small cell lung cancer *in vivo* xenograft mouse model (112). Interestingly first *in vitro* investigations also show efficiency of antibody-mediated inhibition of CEACAM5/CEACAM1 interaction to reduce CEACAM1-dependent NK cell inhibition in pancreatic and CRC cells (113). This indicates an interaction of different CEA family members in this process (101).

### 4.3 Angiogenesis

Generation of new blood vessels based on preexisting ones, called angiogenesis, promotes cancer growth and progression by providing supply with oxygen and nutrients with progressive cancer size. Therefore, targeting angiogenesis was proposed as anti-cancer therapy. However, in clinical practice anti-angiogenic treatment has not been as effective as expected (114). Recent evidence suggests that this might be due to a pro-angiogenic tumor environment that cannot be modulated by systemic drug application. Therefore, more localized interference with angiogenesis might provide better anti-tumor effects than current therapeutic strategies (115–117).

In this regard, CEACAM1 might be a putative target. CEACAM1 is expressed in microvessels within and in close proximity to tumors but not in larger blood vessels (118). This is of particular interest, since CEACAM1 mediates angiogenesis via enhanced VEGF/VEGFR-2 expression (18, 118). In line with this Gerstel et al. reported a crucial role of CEACAM1 for tumor angiogenesis. Using a murine mammary carcinogenesis model, they found that CEACAM1 deficiency results in vascular instability and alterations in ECM structure (119). Furthermore, CEACAM1 deficiency enhanced the permeability of tumor vasculature due to increased basal Akt kinase and endothelial nitric oxide synthase (eNOS) activities (120). This is in line with the concept that CEACAM1 is required for the establishment of the endothelial barrier (121, 122). Besides endothelial cells CEACAM1 expression in myeloid cells also promoted angiogenesis as shown by bone marrow transplantation experiments in mice (123). In contrast, in two subsequent studies Lu et al. reported that tumor angiogenesis mediated by myeloid cells is negatively regulated by CEACAM1 (124, 125). Furthermore, a study of Muturi et al. suggests that CEACAM1 expression in microvesicles derived from cancer cells might influence cancer signaling on other cells including endothelial cells (126).

### 4.4 Migration and invasiveness

Metastasis accounts for more than 90% of deaths from cancer (127). Migration and invasiveness of cancer cells are hallmarks of cancer metastasis that requires tumor cell dissemination from the primary tumor into different organs (128). Ebrahimnejad et al. showed that phosphorylation of CEACAM1-L at position Tyr488 enhances cell migration and matrix invasion of melanoma cells in an integrin β3-dependent manner (129). This is in line with older findings in endothelial cells demonstrating that CEACAM1 affects cell migration and integrin-dependent signaling (130). Forced expression of CEACAM1 in thyroid cancer cells promoted cell–matrix adhesion, migration and tumor invasiveness via upregulation of cyclin dependent kinase inhibitor 1A (p21) and diminished retinoblastoma protein (Rb) phosphorylation. Since this was accompanied by reduced tumor growth in xenografted mice it argues for a pro-metastatic effect of CEACAM1 rather than an effect on the primary tumor (59). Furthermore, CEACAM1 enhanced migration in CRC cells that was ascribed to increased N-cadherin expression (131). Similar, Ieda et al. reported that CEACAM1-L promotes invasion and migration in CRC cells (20). Based on association studies in human samples and a colony formation assay in soft-agar Yamaguchi et al. suggested that expression of CEACAM1-4S enhances the tumor-initiating property of colorectal cancer cells (21). However, this study lacks further analysis that is mandatory for such a statement. In contrast, Yang et al. reported that the short isoform of CEACAM1 decreased cell migration and invasiveness of breast cancer cells by affecting the balance between matrix metalloproteinase 2/tissue inhibitor of metalloproteinase 2 and E-/N-cadherin expression (132). For multiple myeloma cells, Xu et al. found a decrease in cell migration and invasion by CEACAM1 (71). Besides, a recent study suggests that macrophage-cancer cell interaction via CEACAM1 promotes cancer metastasis. This signaling is mediated by β-catenin that enhances metadherin expression on cancer cell surface. Metadherin in turn signals through CEACAM1 expressed on macrophages to produce the chemokine CCL3 (133).

### 4.5 Apoptosis and chemotherapy resistance

Many cancer cells show deregulation of apoptosis that contributes to chemotherapy resistance (134). Therefore, anti-cancer agents have been developed that prime cancer cells for apoptosis (135). One of the first hints that CEACAM1 might support cellular survival by decreasing apoptosis came from studies investigating non-cancer cells like granulocytes and monocytes. Singer et al. showed that tyrosine phosphorylation of CEACAM1-L reduces apoptosis in granulocytes via an Erk1/2- and caspase-3-dependent pathway (136). This is in line with observations in endothelial cells (137). Later, Yu et al. showed that CEACAM1 decreases apoptosis of human monocytes via phosphatidylinositol 3-kinase- and Akt kinase-dependent survival signaling (138). In contrast, CEACAM1 was shown to promote apoptosis in a murine model of myocardial infarction. This was attributed to upregulation of mitochondrial Bax, increased cytosolic cytochrome C and cleaved caspase-3 (139).

Similar studies investigating the impact of CEACAM1 on apoptosis in cancer cells that include signaling pathway analysis are limited. Using siRNA-mediated knockdown of CEACAM1, Han et al. reported an anti-apoptotic effect of CEACAM1 in HT-29 CRC cells (73). In contrast, Nittka et al. showed that antibody-mediated crosslinking of CEACAM1 resulted in enhanced apoptosis in HT-29 CRC cells (4, 140). Unfortunately, in both studies the underlying CEACAM1-dependent signaling pathways were not investigated. Besides affecting apoptosis under basal conditions, CEACAM1-L was also shown to mediate chemoresistance to the widely used chemotherapeutic agent 5-fluorouracil (5-FU) in CRC cells, whereas CEACAM1-S did not. This suggests that long and short form of CEACAM1 might have opposite effects with regard to cell viability (141).

Similar to Nittka et al., Zaffran et al. observed enhanced apoptosis after treatment of Mel-14 melanoma cells with an anti-CEACAM1 antibody *in vitro*. This pro-apoptotic effect was attributed to an altered p53 expression and SHP1 phosphorylation (4, 142). However, in the same study *in vivo* application of this anti-CEACAM1 antibody had no effect on tumor size in a melanoma model (142). Since they were using the same melanoma cells for *in vitro* and *in vivo* experiments, this shows that the results of *in vitro* antibody treatments have to be interpreted with caution.

Of note, forced expression of the short isoform of CEACAM1 in MCF7 mammary carcinoma cells induced a more regular glandular morphology that was accompanied by apoptosis of the central cells within acini (143). However, this seems to be a more development-specific effect rather than a pro-apoptotic effect in general since peripheral acinar cells were not affected. In addition, a bioinformatics-based study analyzing cisplatin resistance in A549 lung carcinoma epithelial cells indicates that CEACAM1 promotes resistance suggesting an anti-apoptotic effect. Though experimental validation in cell culture is lacking yet (144).

## 5 Clinical intervention studies

Based on pre-clinical data CEACAM1-specific antibodies were proposed for anti-cancer therapy. In addition to their inhibitory effects on tumor cells themselves, anti-CEACAM1 antibodies were also reported to promote anti-tumor activity of several immune cell types (92, 111, 113, 145–147). Finally, since CEACAM1 expression in cancer tissue usually exceeds that in the surrounding healthy tissue by far, anti-CEACAM1 antibodies were suggested to be used in surgery to identify cancer tissues in order to enable complete cancer resection (148, 149).

The CEACAM1-specific antibody CM24, developed by cCAM Biotherapeutics Ltd., has shown efficacy against different types of cancer cells *in vitro* and in *in vivo* models of cancer (150, 151). Originally, the company announced initiation of a clinical phase 1 trial of CM24 in 2015 (152). In the same year Merck acquired cCAM Biotherapeutics (153). However, only one year later further development of CM24 was terminated by Merck due to new data: “During 2016, as a result of unfavorable efficacy data, the company determined that it would discontinue development of the pipeline program” (154, 155). Another company, Purple Biotech, plans to evaluate the same antibody CM24 in a phase I studies in the future (156, 157). Based on Merck’s unfavorable results, upcoming clinical studies using the CM24 antibody need to be regarded with caution.

However, the hitherto results do not prove the inefficacy of targeting CEACAM1 in anti-cancer therapy. Rather they suggest that this particular antibody might not be suitable for this purpose.

This is further illustrated by a report from McLeod et al. (158). They showed that another anti-CEACAM1 antibody (Cc1) facilitated binding of soluble CEACAM1 to CEACAM1 expressing cells instead of blocking CEACAM1-dependent signaling as expected. Furthermore, Nakajima et al. suggested that anti-CEACAM1 antibodies may activate CEACAM1-dependent signaling eventually by cross linking of CEACAM1 (78). Thus, antibodies intended to block CEACAM1 signaling in cancer cells probably may induce the opposite effect depending on antibody design. Similar concerns may apply to another anti-cancer strategy. Based on CEACAM1 upregulation, anti-CEACAM1 antibodies are suggested to mark cancer cells for immune cell-based killing. However, beside cancer cells a variety of cells express CEACAM1 depending on activation state and age (1, 2, 18, 159). Hence, this strategy may provoke serious side effects.

In conclusion, it is inevitable to extensively characterize anti-CEACAM1 antibodies that are intended to be used in clinical trials. Then it is reasonable to assume that novel anti-CECAM1 therapeutics might improve survival of cancer patient in the future (112).

## 6 Conclusion and future perspectives

Despite some conflicting findings, the vast majority of clinical studies supports the view that CEACAM1 promotes cancer progression and metastasis in humans. Regarding the interpretation of the results of clinical trials, some aspects have to be considered. First, specificity of anti-CEACAM1 antibodies might be compromised due to cross-reactivity with other CEACAM family members. Second, despite some evidence for possible adverse functions in cancer biology the impact of different CEACAM1 splice variants is still largely unknown.

One of the greatest unresolved issues is how CEACAM1 affects intra- and intercellular signaling since there is no systematic analysis of CEACAM1-dependent signaling in cancer yet. Closing this gap would be an important step to understand the mechanisms that underlie CEACAM1-mediated effects in cancer. This also might reveal other signaling pathways and targets that have not been linked to cancer so far and that could become even more promising targets in cancer therapy than CEACAM1 itself.

In addition, the accumulating evidence of CEACAM1’s repressing function on anti-cancer activity by the immune system makes it an attractive therapeutic target similar to established immune checkpoint inhibition. It is still speculative if such a combined targeted inhibition including CEACAM1 might overcome the limitations of immunotherapies against solid tumors.

## Author contributions

LG: Conceptualization, Writing – original draft, Writing – review & editing. UR: Visualization, Writing – review & editing. GB: Writing – review & editing. VP: Writing – review & editing. SE: Writing – review & editing. FK: Conceptualization, Funding acquisition, Project administration, Supervision, Writing – original draft, Writing – review & editing.
